# Microscopic View of Defect Evolution in Thermal Treated AlGaInAs Quantum Well Revealed by Spatially Resolved Cathodoluminescence

**DOI:** 10.3390/ma11061049

**Published:** 2018-06-20

**Authors:** Yue Song, Ligong Zhang, Yugang Zeng, Li Qin, Yinli Zhou, Yongqiang Ning, Lijun Wang

**Affiliations:** 1 State Key Laboratory of Luminescence and Applications, Changchun Institute of Optics, Fine Mechanics and Physics, Chinese Academy of Sciences, Changchun 130033, China; songyue891216@163.com (Y.S.); qinl@ciomp.ac.cn (L.Q.); 18844074562@163.com (Y.Z.); ningyq@ciomp.ac.cn (Y.N.); wanglj@ciomp.ac.cn (L.W.); 2 Daheng College, University of Chinese Academy of Sciences, Beijing 100049, China

**Keywords:** AlGaInAs quantum well, metal organic chemical vapor deposition, cathodeluminescence, thermal treatment

## Abstract

An aluminum gallium indium arsenic (AlGaInAs) material system is indispensable as the active layer of diode lasers emitting at 1310 or 1550 nm, which are used in optical fiber communications. However, the course of the high-temperature instability of a quantum well structure, which is closely related to the diffusion of indium atoms, is still not clear due to the system’s complexity. The diffusion process of indium atoms was simulated by thermal treatment, and the changes in the optical and structural properties of an AlGaInAs quantum well are investigated in this paper. Compressive strained Al_0.07_Ga_0.22_In_0.71_As quantum wells were treated at 170 °C with different heat durations. A significant decrement of photoluminescence decay time was observed on the quantum well of a sample that was annealed after 4 h. The microscopic cathodoluminescent (CL) spectra of these quantum wells were measured by scanning electron microscope-cathodoluminescence (SEM-CL). The thermal treatment effect on quantum wells was characterized via CL emission peak wavelength and energy density distribution, which were obtained by spatially resolved cathodoluminescence. The defect area was clearly observed in the Al_0.07_Ga_0.22_In_0.71_As quantum wells layer after thermal treatment. CL emissions from the defect core have higher emission energy than those from the defect-free regions. The defect core distribution, which was associated with indium segregation gradient distribution, showed asymmetric character.

## 1. Introduction

An aluminum gallium indium arsenic (AlGaInAs) material system is crucial for semiconductor diode lasers emitting at 1.3–1.55 μm, which has been widely used in optical fiber communications and photonic integrated circuits (PICs) owing to its advantages of high-speed operation, large gain, and external quantum efficiency [1,2,3]. A zinc-blended AlGaInAs compressive strained quantum well (QW) has substantially better electron confinement and material gain performance than the commonly used InGaAsP system, due to a larger conduction band offset [4,5]. For 1.3-μm AlGaInAs multi-quantum wells (MQWs) ridge waveguide lasers, the thermal resistance was found to be 73.6 and 43.5 K/W for junction-up and junction-down mounting, respectively [6]. However, the thermal stability of a quaternary material system is in general worse than that of a binary or ternary system, especially when they are used in diode lasers [7]. Since a thermal process tends to cause the interdiffusion of atoms and defect propagation in quaternary material, which can affect the performances of devices, high temperature stability is somewhat problematic for quaternary AlGaInAs QW-based laser diodes [8]. Micro-cathodoluminescence (CL), excited by a high-energy focused electron beam, is widely applied in detecting alloy fluctuations and structural defects, such as threading dislocations and stacking faults [9,10]. Compared to photoluminescence (PL) excited by light, micro-CL offers a nanometer spatial resolution of emission spectra for semiconductor materials [11]. Therefore, spatially resolved CL is a good candidate to analyze the structural and optical properties of AlGaInAs QWs. For III-V group semiconductors containing indium (In), the segregation of the In atom is always an issue. Fabien C-P. Massabuau et al. suggested that the segregation of In atoms at dislocations influenced the carrier recombination process in InGaN alloys [12]. Manh-Ha Doan et al. reported that the inhomogeneity of In composition in the InGaN/GaN MQW active layer, which led to a potential fluctuation for carrier localization, was critical for the emission efficiency. The high-energy emission shown in CL was caused by the reduction of In composition in the vicinity of the dislocation core [13]. K. Muraki et al. illustrated that the blue shift of transition energy compared to the calculation for a perfectly square quantum well potential was related to the segregation of In atoms in InGaAs QWs [14]. Besides, corroborated experimental and theoretical studies showed that carrier localization induced by indium (In) atoms is much stronger than carrier localization from gallium (Ga) or aluminum (Al), owing to the higher mobility of In atoms [15,16,17]. For quaternary AlGaInAs material, the diffusion process of In atoms under high temperature is still not clear due to its complex structure. The analysis of structural and optical properties under high temperature is very helpful for further studying the degradation mechanism and improving the high temperature stability of AlGaInAs-based devices. In this paper, high-resolution X-ray diffraction (HR-XRD) and scanning electron microscope-cathodoluminescence (SEM-CL) technology were applied to demonstrate the lattice characteristics and composition of AlGaInAs QW affected by thermal annealing, and attempt to gain an insight into the behavior of In atom segregation process in quaternary AlGaInAs alloy.

## 2. Materials and Methods

**Figure 1:**
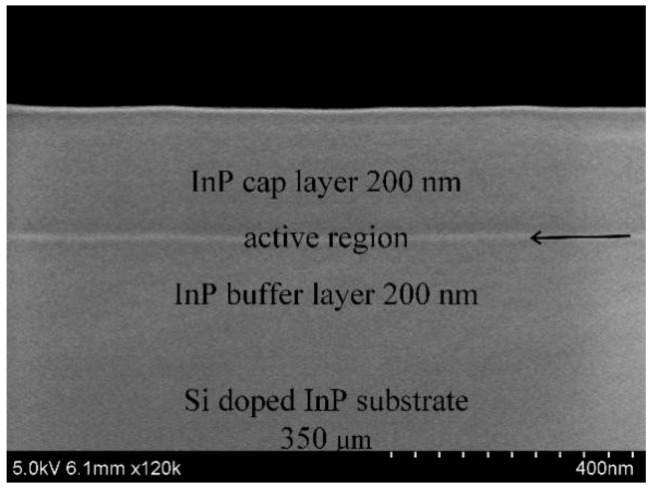
Cross-section scanning electron microscope (SEM) image of Al_0.07_Ga_0.22_In_0.71_As quantum well (QW), as grown.

A three-layer AlGaInAs quantum well sandwiched by a 200-nm indium phosphide (InP) cap layer and a 200-nm buffer layer was grown on a 350-μm thick Si-doped (001) InP wafer by the AIXTRON RF 200/4 Metal Organic Chemical Vapor Deposition (MOCVD) system (Herzogenrath, Germany). The three-layer quantum well active region was composed of a six-nm Al_0.07_Ga_0.22_In_0.71_As quantum well sandwiched between two 10-nm Al_0.225_Ga_0.285_In_0.49_As barrier layers. The morphology of AlGaInAs QW as grown was characterized in a cross-section view by S-4800 scanning electron microscopy (SEM; Hitachi, Tokyo, Japan), as shown in Figure 1. High-purity metal organic (MO) sources including trimethylaluminum (TMAl), trimethylgallium (TMGa), and trimethylindium (TMIn) were used for column III sources. Hydrogen arsenide (AsH_3_) and phosphorus hydride (PH_3_) were used as column V sources. The V/III ratio was 50 for QW and 150 for InP, respectively. Firstly, PH_3_ was imported into the horizontal planetary reactor to prevent the surface decomposition of InP when the substrate was heated to 250 °C. The temperature was gradually increased to 700 °C afterwards, and remained in deoxidation treatment for 10 min. Successively, the temperature was cooled down to 650 °C. MO sources with a proper flux ratio for different sources were in sequence introduced into the reactor chamber for the growth of materials with a growth rate of about 1 μm/h. The center zone of the prepared epitaxial wafer was divided into 15 pieces, which were classified into three groups composed of five pieces. Two groups of AlGaInAs QW samples were thermally treated at 170 °C for 2 h and 4 h, respectively. The room temperature PL spectra of six randomly sampled points were collected for each piece, and all of the experimental pieces underwent PL measurement before thermal treating to estimate the homogeneity of sample and experimental error. Photoluminescence (PL) spectra were measured on a Triax 550 monochromator (Horiba, Kyoto, Japan) excited by an 808-nm line of the continuous-wave semiconductor laser with a fixed power excitation of 12.5 W/cm^2^. The PL spectral resolution was about 0.5 nm. Room temperature radiative decay was detected on a fluorescence spectrometer FL920 (Edinburgh Instruments Ltd., Livingston, UK) equipped with a time correlated single-photon counting technique accompanied by a R5509 fast time respond infrared photomultiplier (PMT; Hamamatsu Photonics, Hamamatsu, Japan). Emission was monitored at the maximum emission wavelength under an excitation of a 635-nm picosecond pulse diode laser (EPL-635; Edingburgh Instruments Ltd., Livingston, UK) with a pulse duration of 180 ps, and a repetition frequency of 1 MHz. The lattice characteristics of QWs were measured by the D8-Discover film X-ray diffraction (CuKα 40KeV, 30mA; Bruker, Billerica, MA, USA). The microstructural properties were characterized by scanning electron microscope-cathodoluminescence (SEM-CL), which was realized with an Attolight Alalin Chronos 4027 system (Attolight AG, Écublens, Switzerland) coupled with Jobin Yvon spectrometer (iHr320; Horiba, Kyoto, Japan) and a Synapse charge coupled device (CCD) camera (Horiba, Kyoto, Japan) at 25 K in top view. The spatial resolution of CL measurements was less than 10 nm. Hyperspectral CL maps resolution was 128 × 128 pixel (integration time was 1 ms/pixel). The electron beam energy was 10 keV. Each spectrum was averaged over each scanned area. The CL spectral resolution was about 0.5 nm.

## 3. Results and Discussion

Each sample in this experiment before annealing revealed a PL emission band centered at 1477 nm corresponding to the Al_0.07_Ga_0.22_In_0.71_As QW layer. The PL spectrum of AlGaInAs QW is similar to that reported by Li, X.J. [18]. The samples as grown (before thermal treatment) were quite homogeneous within the resolution limit of the Triax 550 spectrometer. For ternary InGaAs-based laser diodes, an increment of temperature (about 200 °C) occurred in the active region after a long period of current injection. Some hotspots presented in the active layer when the local temperature reached 130 °C [19]. In this experiment, we chose 170 °C annealing to estimate the structural and optical properties changes caused by temperature.

**Figure 2:**
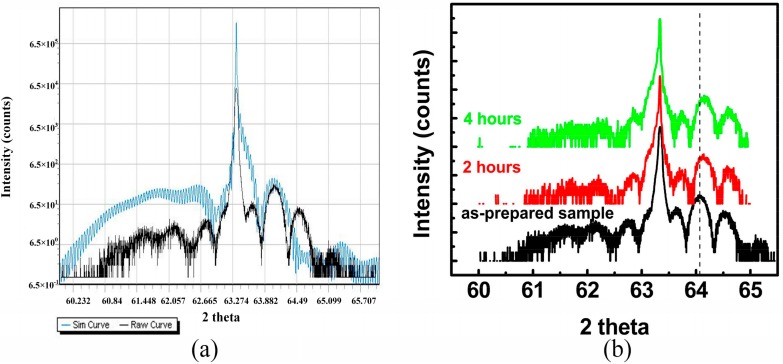
(**a**) Diffraction pattern of the sample as grown compared with a simulated curve. The blue curve is the simulation of the sample as grown. (**b**) Intensity (counts)-2θ scans of high-resolution X-ray diffraction (HR-XRD) patterns of Al_0.07_Ga_0.22_In_0.71_As QW thermally treated at 170 °C with different durations.

The crystallization and lattice properties of strained Al_0.07_Ga_0.22_In_0.71_As QW samples were investigated by high-resolution X-ray diffraction (HR-XRD). Figure 2 illustrates the HR-XRD patterns along the (004) orientation of Al_0.07_Ga_0.22_In_0.71_As QW treated with various annealing durations at 170 °C. The diffraction patterns were dominated by the QW diffraction and InP (004) diffraction peaks. The peak at 63.345° (for two theta) was originated from the InP layer and interference patterns located at the side vicinity were from AlGaInAs QW [20]. Interference fringes distributed over a wide angle range were affected by the composition and thickness of the Al_0.07_Ga_0.22_In_0.71_As quantum well and Al_0.225_Ga_0.285_In_0.490_As barriers. XRD analysis software Leptos (Bruker, Billerica, MA, USA) was employed to simulate the diffraction pattern based on the composition and thickness of each layer of the samples. Lattice qualities of the samples were assessed by comparison with the simulating curve and diffraction pattern. The simulation of diffraction for the sample as grown was drawn in a blue curve, as shown in Figure 2a. The diffraction pattern of the sample as grown was highly consistent with the simulation curve, the main peaks of the multi-order satellite peaks in the diffraction pattern coincided well with the simulation curve obtained from Leptos. For the sample thermally treated at 170 °C for 2 h, the diffraction pattern was similar to that of the sample as grown, but the QW’s multi-order satellite peaks slightly moved to larger 2θ degrees (the shift was about 0.091 degree), as shown in Figure 2b. While, for a sample that was heat-treated for four hours, the QW’s multi-order satellite peaks continued to largely shift and became more obvious (almost 0.096 degree). Due to a larger ionic radius of In (1.55 Å) in comparison with Ga (1.3 Å) and Al (1.25 Å), indium composition decreased after heat treatment, leading to a lattice deformation [21]. Furthermore, the intensity of multi-order satellite peaks on the left side of an InP diffraction peak decreased obviously, indicating the deteriorative tendency of lattice quality after annealing [22].

**Figure 3:**
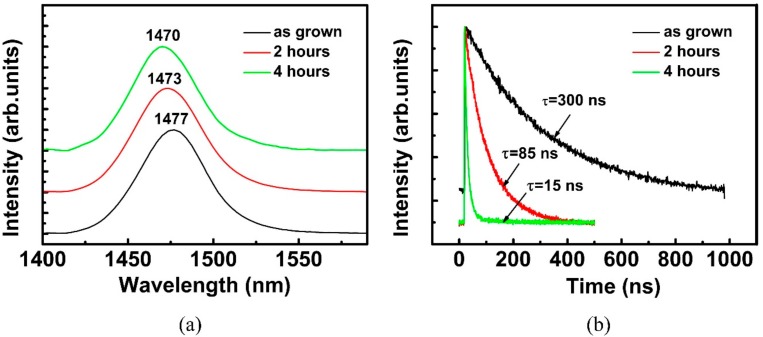
(**a**) Room temperature (300 K) photoluminescence spectra of Al_0.07_Ga_0.22_In_0.71_As QW thermally treated with different durations at 170 °C. (**b**) Time-resolved photoluminescence (PL) spectra of sample as grown, after 2 h and 4 h (respective emission wavelength centered at 1477 nm, 1473 nm, and 1470 nm).

Figure 3a shows the room temperature PL spectra of Al_0.07_Ga_0.22_In_0.71_As QW with different thermal treatment durations. The spectrum of sample as grown revealed a single-band emission, and the emission peak was centered at 1477 nm. The PL emission peak showed an obvious blue shift ranging from 1477 to 1470 nm with annealing duration increasing. This phenomenon could be attributed to a decrease in the In concentration with the temperature [23]. The full width at half maximum (FWHM) value subtle increased simultaneously from 25.8 meV for AlGaInAs QW as grown to 26.7 meV for 4 h of thermal treatment. Figure 3b shows the emission decay traces for samples as grown, which were thermally treated at 170 °C for 2 h and 4 h, respectively. All of the decay curves followed a single-exponential function. The long decay time of the sample as grown (300 ns) indicated a high-quality crystal epitaxial growth of QW. However, faster PL decay was observed in the sample that was annealed after 4 h (15 ns). The effective decay time *τ* of QW excitons is given by [24]:

(1) $$1/\tau =1/\tau_{r}+1/\tau_{nr}$$

where *τ_r_* and *τ_nr_* are the exciton radiative and non-radiative lifetimes, respectively. *τ_r_* is related to the energy band width corresponding to the transition of irradiation and carrier scattering in alloys, and *τ_nr_* is influenced by the non-radiative recombination channel. For a sample treated at 170 °C for 4 h, the value of effective decay time *τ* decreased by 95%, at least in comparison with that as grown. However, the increase of emission line width was just 1.05 times larger than the sample as grown. The widening of the emission band did not account for the decrement of decay time, implying that the more severe decrease of PL lifetime was attributed to the influence of non-radiative recombination. Since the increment of dislocation density led to the deterioration of lattice quality, the non-radiative recombination process increased significantly while the quality of lattice degraded, leading to the decrease of PL lifetime [24,25]. Thus, we deduce that dislocation density increments and significant alloy fluctuations occurred in the Al_0.07_Ga_0.22_In_0.71_As QW layer after a long time of thermal treatment.

**Figure 4:**
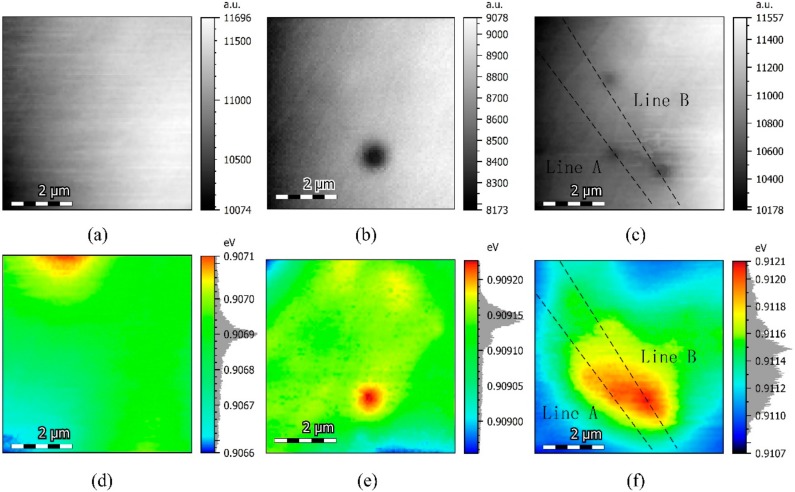
Cathodoluminescent (CL)-integrated intensity mappings of sample (**a**) as grown (**b**) 2 h (**c**) 4 h at the emission energy of Al_0.07_Ga_0.22_In_0.71_As QW at 25 K. Corresponding absolute CL peak energy shift mappings of (**d**) as grown (**e**) 2 h (**f**) 4 h.

In order to get insight into the microstructure variation induced by thermal treatment, SEM-CL was applied on an AlGaInAs QW to probe the spatial distribution of the CL emission wavelength. Electron–phonon interaction seriously affected the emission at room temperature. Since electron–phonon interaction could be repressed at a low temperature, the CL signal increased dramatically [26]. Moreover, carriers were demonstrated to be frozen at low temperature, and could be trapped on the first shallow localization center encountered; no significant spectral shift appeared at the temperature range below 50 K [27,28]. Therefore, we employed spatially resolved cathodoluminescence (SRCL) at 25 K to analyze the structural and optical changes induced by annealing. The SRCL-integrated intensity and corresponding peak energy shift mappings of Al_0.07_Ga_0.22_In_0.71_As QWs thermally treated with different durations are illustrated in Figure 4. Each mapping is composed of 5200 CL emission spectra. The SRCL-integrated intensity mapping of the Al_0.07_Ga_0.22_In_0.71_As QW as grown presented slightly statistical fluctuations due to the weak energy variations of the confinement potential. No obvious dark spot was observed in Figure 4a. Figure 4d is the absolute peak energy shift mapping corresponding to the CL intensity in Figure 4a. The SRCL peak variation between maximum and minimum emission energy was about 0.5 meV. The grey histogram at the right-hand side of each peak energy shift mapping reflected the density distribution of the SRCL emission peak. The density distribution of the CL emission peak energy obeyed an approximate Gaussian distribution centered at 1367.3 nm in the sample, as grown. The emission energy was spatially uniform within the resolution limit of the technique.

Figure 4b illustrates the top-view CL-integrated intensity mapping of the Al_0.07_Ga_0.22_In_0.71_As QW thermally treated at 170 °C for 2 h. Statistical fluctuation and an obvious dark spot can be found in Figure 4b. Generally, dark spots on the bright matrix observed in CL-integrated intensity mapping were caused by the presence of extended lattice defects such as dislocations [29]. However, unlike the study that reported that dark spots in CL behave as non-radiative recombination centers [30], these dark spots in our samples did not completely annihilate the CL signal, and the CL emission energy at the dark spot was higher than that of the surroundings. The significant reduction of luminescence in the dark spot vicinity should be attributed to the drastic decrease of the carriers’ population in these dark spots due to the energy band elevation. Moreover, since the AlGaInAs QW layer was covered by a 200-nm InP cap layer, the spatial resolution of AlGaInAs QW intensity mapping would be reduced. Therefore, the true size of the defect region was smaller than the dark spot shown in Figure 4b. Figure 4e depicts that the absolute CL peak energy shift mapping of Al_0.07_Ga_0.22_In_0.71_As QW annealed after 2 h, corresponding to the CL intensity in Figure 4b. The defect localization area could be identified as a dark red spot surrounded by a bright halo in the energy shift mapping. The bright halo exhibited a lower energy shift relative to the center of the dark red spot. The carrier localization induced by In atoms is much stronger than carrier localization from Ga or Al; therefore, the red spot might be related to carrier localization caused by the segregation of In atoms [31,32,33]. Furthermore, the density distribution of the CL emission peak was distinctly asymmetric, including the most probable emission peak at 1363.9 nm accompanied with a small distribution peak at the lower energy side.

**Figure 5:**
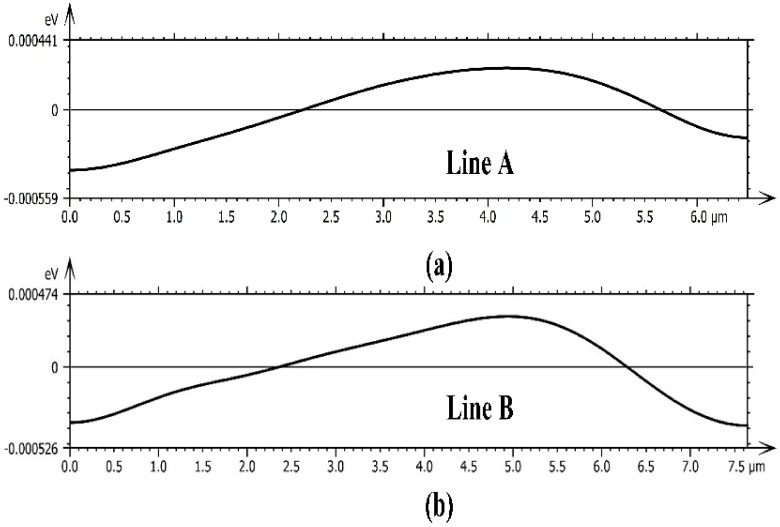
Emission energy change profiles of line A (**a**) and B (**b**).

Figure 4c shows the CL-integrated intensity mapping of Al_0.07_Ga_0.22_In_0.71_As QW thermally treated at 170 °C for 4 h. More dark spots were revealed in CL mapping compared with that of the sample with a shorter duration of thermal treatment. The crimson spots corresponded to the high energy emission from the center of dark spots, and these spots exhibited a 2.1-nm blue shift relative to the bright area shown in Figure 4f. The spatial variation of the CL emission peak was disordered. Besides, the CL peak density distribution in the energy was quite unconformity. The obvious increasing of the dark spots’ density and significant alloys fluctuations in the Al_0.07_Ga_0.22_In_0.71_As QW layer after longer annealing times is probably the dominant factor of faster PL decay. Generally, the modification of transition energies was attributed to changes in the composition [34]. Through monitoring the energy shift changes, we are able to deduce the alloy composition variation. The emission peak energy distribution gradient near the dark spot with highest peak energy was not coincident in all of the directions. As shown in Figure 4f, it was quite gentle along with the dense region of defect, but rather steep on the border near the bright region. The emission peak energy shift gradient profile of line A and B labeled in Figure 4c,f are plotted as Figure 5. The changes in emission energy correspond to the cut lines ranging from the upper left corner to the lower right corner. The 0 position corresponds to the average energy level of each line. This gave a qualitative illustration of the energy diffusion near the dark spots in the QW layer.

**Figure 6:**
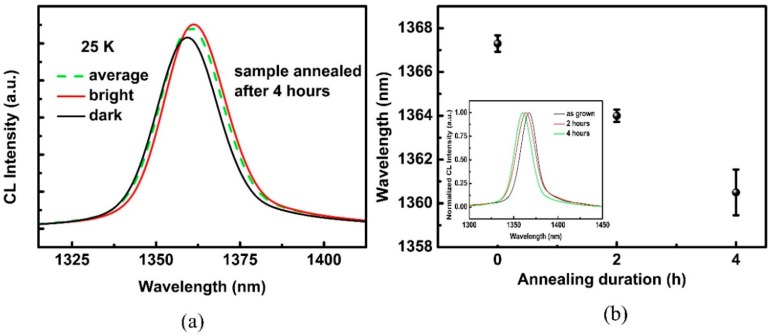
(**a**) Most probable emission CL spectra in the mapping (dashed line) and spatially resolved cathodoluminescence (SRCL) spectra with maximum and minimum emission energy (solid lines) for sample annealed after 4 h. (**b**) The SRCL distribution range for Al_0.07_Ga_0.22_In_0.71_As QW with different thermally treated durations. Inserted illustration shows the most probable emission CL spectrum of each sample.

Figure 6a shows the CL spectra of the most probable emission within the mapping area (dash line), and the SRCL spectra from the defect region and the defect-free region (solid line) for the sample annealed after 4 h. Each CL spectrum was characterized by a single emission band. The SRCL spectra showed that emissions centered at 1361.6 nm from the defect-free region had lower energy than that from dark spots (1359.5 nm at defect region). Owing to the larger mobility of the In atom and its segregation led to a blue shift of the transition energy [35,36], we can deduce that the surrounding matrix was richer in In than the dark spots, and these defects might be caused by the segregation of In atoms. Additionally, the emission energy distribution range of the SRCL spectra for Al_0.07_Ga_0.22_In_0.71_As QW with different thermally-treated durations is shown in Figure 6b. The inserted illustration is the most probable emission spectrum of each sample. As annealing duration increased, the peak of most probable emission spectrum showed a blue shift ranging from 1367.3 nm to 1360.4 nm. The tendency is in accordance with that of PL detected at room temperature. The gap distance between the adjacent rods in Figure 6b indicates the peak distribution range of SRCL. As can be seen, the gap distance changed little between the samples as grown and thermally treated for 2 h at 170 °C, but a pronounced increment occurred in the sample annealed after 4 h. This could be attributed to the dramatic diffusion phenomenon of In atoms after a long annealing time. As the reported reference for the InGaN thin layer [31], the energy ring encircling dislocation had a more pronounced opposite energy shift along different directions, reflecting that the diffusion process of In atoms occurred intra-layer. In our samples, we just observed that the emission shifted to the higher energy occurring at the dark spots, as shown in Figure 4. This implies a weak diffusion of the In atom intra-layer, and a dominant diffusion between interlayers. Additionally, the lattice quality of annealed samples would be deteriorated due to the segregation of the In atom. As can be seen in Figure 2, the intensity of the multi-order QW’s satellite peaks on the left side of the InP main peak decreased obviously. While the quality of lattice degraded, the non-radiative recombination process would be significantly increased, leading to the decrease in the PL lifetime [22].

**Figure 7:**
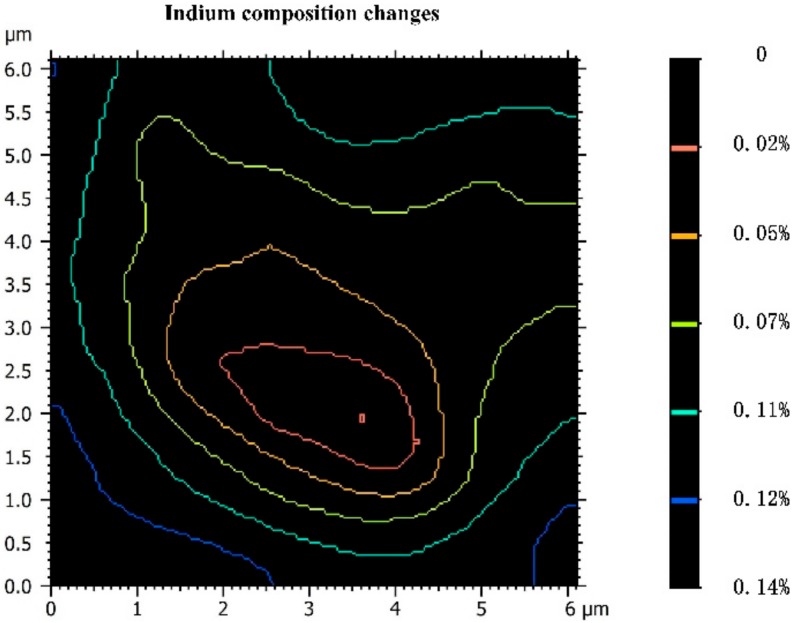
Simulation of In composition increment in the vicinity of the dark spot (the central point corresponds to the maximum degree of the In segregation point).

Empirical band gap relation for the AlGaInAs alloy was taken into account to calculate the composition variation in the QW layer [37,38,39,40]. In order to meet the requirement of the material to remain stoichiometric, since Al did not diffuse significantly, In diffusion was considered to maintain the structure of this material [17]. Hence, the asymmetric cathodoluminescence was associated with the In atoms’ segregation diffusion gradient distribution. Figure 7 shows In composition changes near the dark spots. The central point corresponds to the maximum degree of the In segregation point. For the 4-h thermally treated sample, the In atoms’ composition changes surrounding the dark spot, which had the highest energy, were different along the circumference. One direction of the contour line had a more pronounced positive change in In composition (almost 0.12% in just the 1-μm range), but another direction showed many gradual changes, making the contour line like a bean, partially encircling the dark spot. These results indicated that the In atom diffusion process is asymmetric near the defect core region.

## 4. Conclusions

XRD and spatially resolved SEM-CL have been used to determine the structural and optical changes of Al_0.07_Ga_0.22_In_0.71_As QW annealed with different durations at 170 °C. The PL decay of QW annealed after 4 h exhibited a characteristic decay time that was 20 times shorter than the sample as grown. The SRCL distribution of the AlGaInAs sample annealed after 4 h was disordered. The peak energy corresponding to the defect region had a blue shift, thus indicating the defects contained less In atoms than the surrounding matrix, and the In atom diffusion process was asymmetric near the defect region. This is probably attributed to the dramatic segregation of In atoms between interlayers a after long annealing time.
